# Patient Repayment of US Hospital Bills From 2018 to 2024

**DOI:** 10.1001/jamahealthforum.2025.2284

**Published:** 2025-08-08

**Authors:** Benedic Ippolito, Erin Trish, Erin L. Duffy, Boris Vabson

**Affiliations:** 1 American Enterprise Institute, Washington, DC; 2 Schaeffer Center for Health Policy & Economics, University of Southern California, Los Angeles; 3 Harvard Medical School, Harvard University, Boston, Massachusetts

## Abstract

**Importance:**

Patient cost sharing liability has risen in recent years, increasing the costs associated with care for patient households and imposing collections challenges for hospitals and clinicians.

**Objective:**

To measure patient repayment of cost sharing over recent years, and how that varies across patient, hospital, and service characteristics.

**Design, Setting, and Participants:**

This cross-sectional study of billing and payment data from a revenue cycle management company examined patient accounts at 217 US hospitals from 2018 to 2024. Participants included 24.5 million and 6.2 million patient episodes of care with positive patient out-of-pocket liability for individuals with private insurance and Medicare Advantage, respectively.

**Main Outcomes and Measures:**

Percentage of owed patient cost sharing actually paid among patients with private insurance and Medicare Advantage measured over time, for inpatient and outpatient care and by bill size.

**Results:**

Across the full sample of 217 US hospitals (30.7 million patient episodes), mean (SD) patient liability per person, including those with no liability, was higher for individuals with private insurance ($375.41 [$51.55]) than those with Medicare Advantage ($172.50 [$14.84]). Consistent with high-deductible plan design with annual resetting, mean patient liability was higher for visits in January than December, particularly among the privately insured (eg, mean [SD] patient liability for visits among the privately insured in January: $479.44 [$29.21] vs December: $321.63 [$14.29]). Prior to the COVID-19 pandemic (January 2018-February 2020), mean repayment rates were 53.9% and 54.0% for patients with private or Medicare Advantage insurance, respectively, and repayment rates declined in more recent years. Across the entire sample, patients with private or Medicare Advantage insurance paid either 0% or 100% of their owed cost sharing in 92.2% and 94.1% of cases, respectively. Repayment rates varied by bill size with lower repayment rates on the largest bills and the smallest bills, and higher repayment rates on midsized bills.

**Conclusions and Relevance:**

This cross-sectional study found that patient cost sharing repayments were incomplete and have fallen in more recent years, which result in both medical debts for patients and collections shortfalls for hospitals and clinicians. These findings suggest that changes to insurance plan design or the treatment of medical debt are among several factors that may contribute to observed results; if declines in cost sharing repayment continue, hospitals and clinicians may increasingly seek payment of cost sharing ahead of service, when allowable.

## Key Points

**Question:**

How has patient repayment of owed cost sharing changed over recent years, and how does it vary with patient, clinician, and service characteristics?

**Findings:**

This cross-sectional study of patient accounts at 217 US hospitals from 2018 to 2024 found that prior to the COVID-19 pandemic (January 2018-February 2020) mean repayment rates were approximately 54% and declined in recent years. Repayment rates varied by bill size with lower repayment rates on the smallest and largest bills, and higher repayment rates on midsized bills.

**Meaning:**

These findings suggest that changes to insurance design and consumer protections removing medical debt from credit reporting may contribute to recent trends of incomplete and decreasing repayment rates, which contribute to medical debts and hospital collection shortfalls.

## Teaser

This cross-sectional study measures US patient repayment of cost sharing from 2018 to 2024, and how that varies across patient, hospital, and service characteristics.

## Introduction

Patient cost sharing has risen over time and become increasingly concentrated in deductibles.^1^ Despite comprising a meaningful portion of the total payments owed to hospitals and physicians, evidence suggests that a large amount goes unpaid. Indeed, approximately 13.7 million adults with insurance report holding at least $250 in medical debt,^2,3^ which likely reflects only a portion of the overall unpaid cost sharing. Industry reports also suggest hospitals collect substantially less than what insured patients owe.^4^

Incomplete repayment of cost sharing is likely affected by both the financial circumstances of patients and institutional features of health care billing. Notably, most cost sharing is collected after services have been delivered. In some cases, this time lag is substantial. This can create confusion and may lessen the impetus for some patients to pay since services cannot typically be recalled. Efforts to collect owed cost sharing are also administratively costly, which may weaken hospitals’ and clinicians’ incentives to spend resources doing so. Consistent with this, consumer advocacy groups often encourage consumers to treat medical debt as a lower priority form of debt.^5^

Given the evolving nature of insurance coverage and recent policy changes, it has become increasingly important to understand the extent to which cost sharing is paid and how that varies along key dimensions. The shift toward deductibles means that patient financial liability is more concentrated, resulting in larger single bills compared to coverage with otherwise similar insurance that has copays or coinsurance applied to all utilization throughout a year.^6^ This also means that patient cost sharing is likely to represent a larger share of total payments to hospitals and clinicians when patients are in the deductible phase of their coverage.

Ongoing changes to the treatment of medical debt have the potential to alter repayment rates as well. Starting in July 2022, many unpaid medical bills started being removed from credit reports from the 3 largest credit reporting agencies in the country.^7^ The Consumer Financial Protection Bureau issued a rule in January 2025 that bans all medical debts from credit reports.^8^ This attenuates one way in which not paying medical bills can harm consumers, potentially reducing repayment rates.

There is very limited empirical research that examines how often patient cost sharing is paid. Data availability presents a major challenge for research on this topic because very few sources track patient liability and repayments. Insurance claims data, for example, typically includes the amounts the insurer attributes as patient liability—deductible, copay, coinsurance—but does not capture whether those payments are ever collected by health care facilities or clinicians. One study used data from an electronic health record firm in 2015 and found that clinicians collected 81% of cost sharing for small to modestly sized bills associated with ambulatory services, such as ambulatory surgical procedures and office visits.^9^ It is unclear how representative this is of other settings, bill sizes, or more recent years. Industry reports can be suggestive but include little information about methods and often present very narrow snapshots of data.

Our study explores this question in a broader way. We use data from a revenue cycle management company from 2018 to 2024 to explore the rate at which cost sharing is paid across a broad set of hospital and physician services within inpatient and outpatient settings and across hundreds of major health systems nationwide. These data allow us to ask how repayment of cost sharing has changed over recent years and how it varies with patient, hospital, and service characteristics.

## Methods

The University of Southern California institutional review board determined this cross-sectional study exempt from review because it was not human participant research. This report follows the Strengthening the Reporting of Observational Studies in Epidemiology (STROBE) reporting guideline.

### Data Source

Data for this study were obtained from FinThrive, a large revenue cycle management company.^10^ This revenue management company works with hospitals and health systems to manage billing and collection services associated with patient care. Through this work, it obtains data on all hospital billing and payment activities. Unlike standard insurance claims data, which typically include billing and payment information associated with insurers (and generally assumes full repayment of cost sharing), these data also track patient billing and repayment activities.

The data cover patient episodes that occurred between the first quarter of 2018 and the third quarter of 2024. Before additional restrictions, the full sample includes observations with information about patient liability and repayments from 494 hospitals and health systems. Each observation in our data represents a single episode of care and includes information about services provided, place of service, and patient insurer type, as well as financial information like original charges, allowed amounts following contractual discounts off charges, actual payments from insurers, patient liability, and actual patient repayments. The data also include the month and year of visit or discharge for each observation.

The data include all payments associated with these episodes that were made by September 2024 (when our data were produced). A given episode may include multiple individual bills. The sample includes inpatient and outpatient services provided at hospitals as well as professional services from clinicians that bill through the hospital. They exclude bills for professional services not billed through the hospital and care provided by stand-alone facilities that do not bill through a hospital.

Because our analysis focuses on repayment of patient cost sharing, we include individuals with private insurance (including both commercial and individual plans) or Medicare Advantage who are not dually eligibility for Medicaid, because these individuals can face meaningful cost sharing amounts. Medicare Advantage enrollees are identified through detailed textual descriptions of plans (eAppendix in Supplement 1).

We exclude those enrolled in Medicaid and Traditional Medicare (who almost always have secondary insurance) because they face very limited levels of cost sharing from hospitals. Our core outcome of interest is the repayment rate, which is defined as the fraction of patient liability that is recorded as paid by September 2024. We include episodes that occurred no later than the third quarter of 2023 to allow at least 1 year of follow-up for all bills. We also calculate the fraction of patient liability that was paid within 1 year of an episode for a subset of our sample with required data.

We implemented exclusions at the hospital and episode level. We excluded hospitals reporting implausible repayment data, which we conservatively defined as hospitals with mean repayment rates below 5%. At the episode level, we dropped any remaining observations where one of our calculated repayment rates was negative or greater than 1, because these reflect data errors. This removed 6.6% of observations. Supplement 1 outlines this process in greater detail. Additionally, our primary analyses focus on a balanced sample of hospitals appearing in all quarters of our data for the full study period.

### Measures

To measure the magnitude of patient liability over time, we computed the monthly mean amount of cost sharing owed per episode of care for the private and Medicare Advantage patient populations. For each episode of care that had positive patient liability, we measured repayment rate as the proportion of cost sharing owed that was paid by the patient. Then, we computed the mean (unweighted) monthly repayment rate across episodes of care for the private and Medicare Advantage patient populations. We also considered repayment rates by patient age. Finally, we assessed the repayment rate of bills, categorizing bills in $50 increments (eg, $1-$50, $51-$100) across the range from $1 to $2000. Monetary values were inflated to 2024 nominal US dollars using the Consumer Price Index for Urban Consumers (CPI-U).

### Statistical Analysis

Data were analyzed from October 2024 to March 2025. Categorical outcomes are described with frequencies and percentages. Continuous outcomes are described with means and standard deviations. Line graphs are used to display trends over time in liabilities and repayment rates. Scatter plots are used to assess the association between repayment rates and bill size. All statistical analyses were performed using Stata, version 16 (StataCorp LLC).

## Results

Our primary analysis (balanced) sample uses observations from the 217 hospitals that appear in all quarters of our data. When compared with the universe of hospitals in the US, hospitals in our balanced sample are more likely to be urban (87.10% vs 67.86%), slightly less likely to be teaching hospitals (21.66% vs 25.60%), and tend to be larger (29.50% have over 500 beds vs 4.77%) (Table). See the eAppendix in Supplement 1 for discharge weighted characteristics.

**Table.** Sample Characteristics at Hospital Level

Characteristic	Hospitals, No. (%)		
	Balanced sample (n = 217)^a^	Full sample (n = 399)^a^	US sample (equally weighted) (n = 6152)^b^
Urban	189 (87.10)	343 (85.96)	4154 (67.86)
Teaching^c^	47 (21.66)	93 (23.31)	1567 (25.60)
Bed size			
<200	47 (21.66)	112 (28.07)	4742 (77.47)
200-299	69 (31.80)	116 (29.07)	505 (8.25)
300-499	35 (16.13)	61 (15.29)	478 (7.81)
≥500	64 (29.50)	107 (26.81)	292 (4.77)
Missing	2 (0.92)	3 (0.75)	104 (1.70)

^a^ Analysis of hospitals from FinThrive data. See the eAppendix in Supplement 1 for details about data source and construction.

^b^ Data from RAND Hospital Files for calendar year 2023 (data vintage February 2025), which are based on the Healthcare Cost Report Information System from the Centers for Medicare & Medicaid Services. Urban status for this sample is defined as a Rural-Urban Continuum Code greater than 3.

^c^ Teaching hospitals defined as those who are either members of the Council of Teaching Hospitals or have residency programs.

After data restrictions, we observed a total of 24.5 million and 6.2 million episodes with positive patient liability for individuals with private insurance and Medicare Advantage, respectively (68.7% of observations in our sample have positive liability). This corresponds to a mean of 355 500 (private insurance) and 90 400 (Medicare Advantage) episodes per month. Because we use a balanced sample of hospitals, the sample remains fairly consistent across our study period, except for the second quarter of 2020 due to COVID-19. See the eAppendix in Supplement 1 for details on sample size over time.

**Figure 1.**
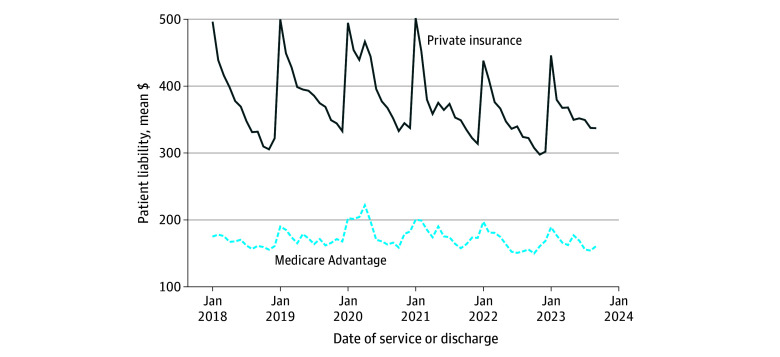
Mean Patient Liability per Episode, by Type of Insurance, 2018-2023 Medicare Advantage category excludes those with dual eligibility for Medicaid. Means include episodes with no patient liability. See eAppendix in Supplement 1 for details about FinThrive data source and construction.

Across our full balanced sample, the monthly mean (SD) patient liability per person per episode, including those with no liability, was higher for individuals with private insurance ($375.41 [$51.55]) than those with Medicare Advantage ($172.50 [$14.84]). Mean (SD) patient liability among the privately insured was approximately 49% higher for episodes in January ($479.44 [$29.21]) than December ($321.63 [$14.29]) across our sample (Figure 1). This difference was approximately 13% for those with Medicare Advantage ($192.35 [$10.02] vs $170.59 [$8.09]). This pattern is similar if we separate inpatient and outpatient episodes (eAppendix in Supplement 1).

**Figure 2.**
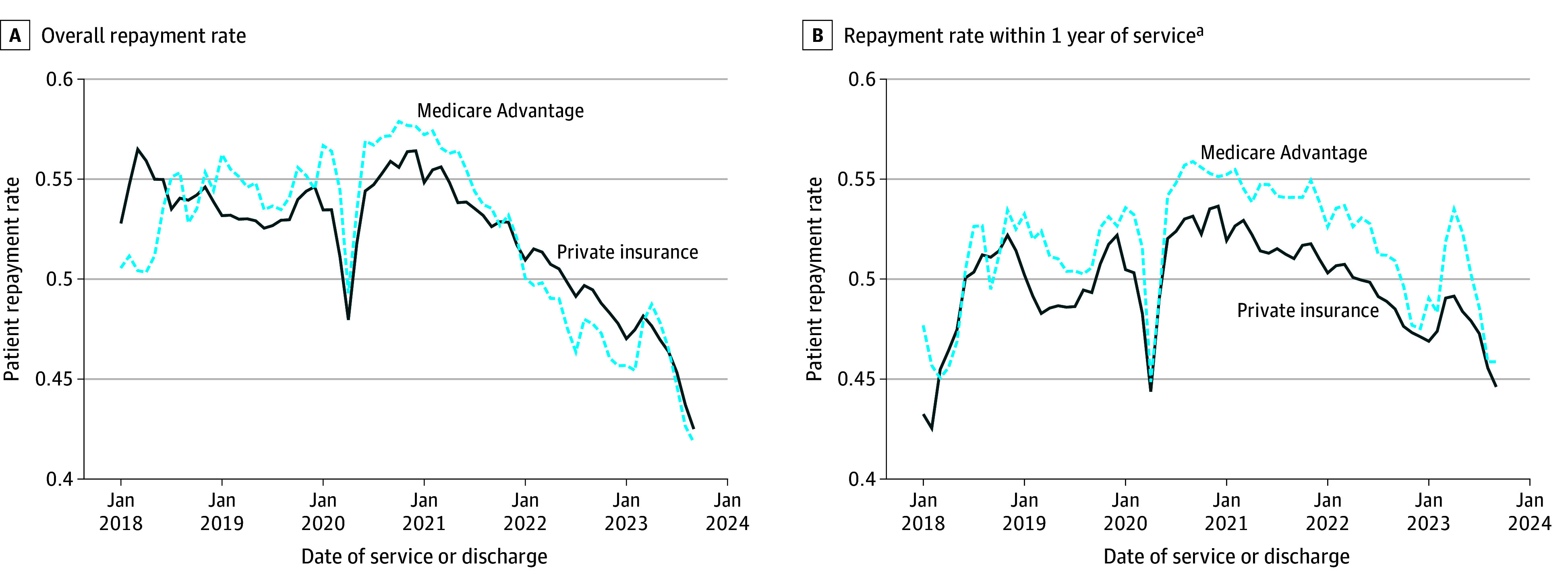
Mean Repayment Rate by Type of Insurance, 2018-2023 Repayment rate is defined as the fraction of patient liability recorded as paid by September 2024. Medicare Advantage (MA) category excludes those with dual eligibility for Medicaid. Sample restricted to episodes with positive patient liability. See eAppendix in Supplement 1 for details about FinThrive data source and construction. ^a^Includes subset of observations with reliable transaction-level information.

Among episodes with positive patient liability, prior to the COVID-19 pandemic (January 2018-February 2020), mean repayment rates were 53.9% and 54.0% for patients with private or Medicare Advantage insurance, respectively (Figure 2A). We observed declining repayment rates for both groups in more recent years. Among the privately insured, mean repayment rates for visits in 2023 were 14.3% (or 7.7 percentage points) lower than in 2021 (46.1% vs 53.8%). Among those with Medicare Advantage, repayment rates fell by 16.8% (or 9.2 percentage points) over this time (45.7% vs 54.9%). These results are similar if we use all hospitals that ever appear in our sample rather than a balanced sample of those that appear in all quarters (eAppendix in Supplement 1).

Because newer bills have had less time to be repaid, and this may bias time trends in repayment measures toward higher repayment rates in earlier bills, we also calculated the fraction of patient liability paid within a year of visit or discharge for the portion of our sample with required data available (Figure 2B). This creates a consistent measure because all visits in our sample have had at least 1 year to be paid, and it does not account for repayments made beyond that time period for any observations. Among the privately insured, mean 1-year repayment rate was 8.3% lower (or 4.3 percentage points) for visits from 2023 compared with those from 2021 (47.4% vs 51.7%). The one-year repayment rate for those on Medicare Advantage decreased 9.2% (or 5 percentage points) over this same period (54.5% vs 49.5%).

**Figure 3.**
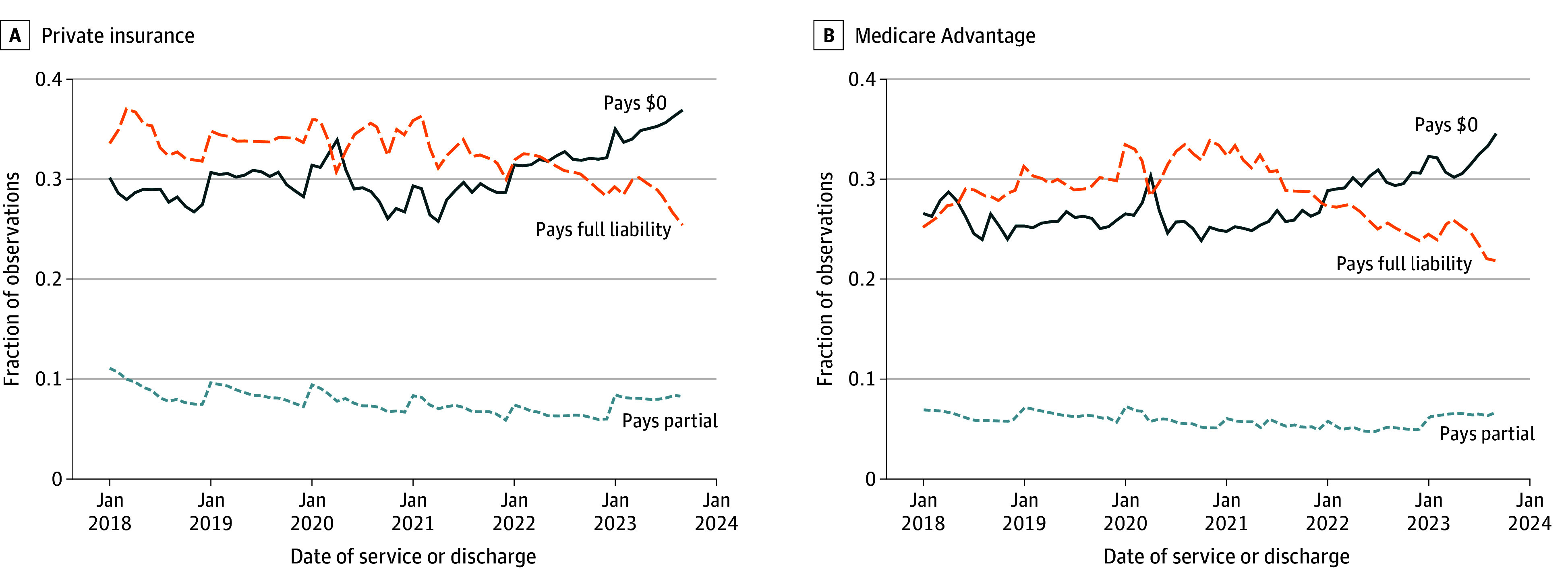
Proportions of Observations Paid in Full, Partially Paid, and Unpaid by Insurance Type This analysis illustrates the fraction of observations where patients pay 100% of patient liability, 0% of patient liability, or any other amount in between. Medicare Advantage category excludes those with dual eligibility for Medicaid. Sample restricted to episodes with positive patient liability. See eAppendix in Supplement 1 for details about FinThrive data source and construction.

Across all years in our sample, patients with private or Medicare Advantage insurance paid either 0% or 100% of their owed cost sharing in 92.2% and 94.1% of cases, respectively (Figure 3). The decrease in repayment rate observed toward the end of our sample is driven by a larger share of patients paying nothing, rather than patients paying a smaller portion of the amount owed on their bill.

Repayment rates vary considerably across age groups, particularly within the privately insured. Repayment rates are lowest for adults aged 20 to 29 years and increase with age thereafter (eAppendix in Supplement 1). In addition, the reduction in repayment rates have been fairly consistent across the different age groups (eAppendix in Supplement 1).

**Figure 4.**
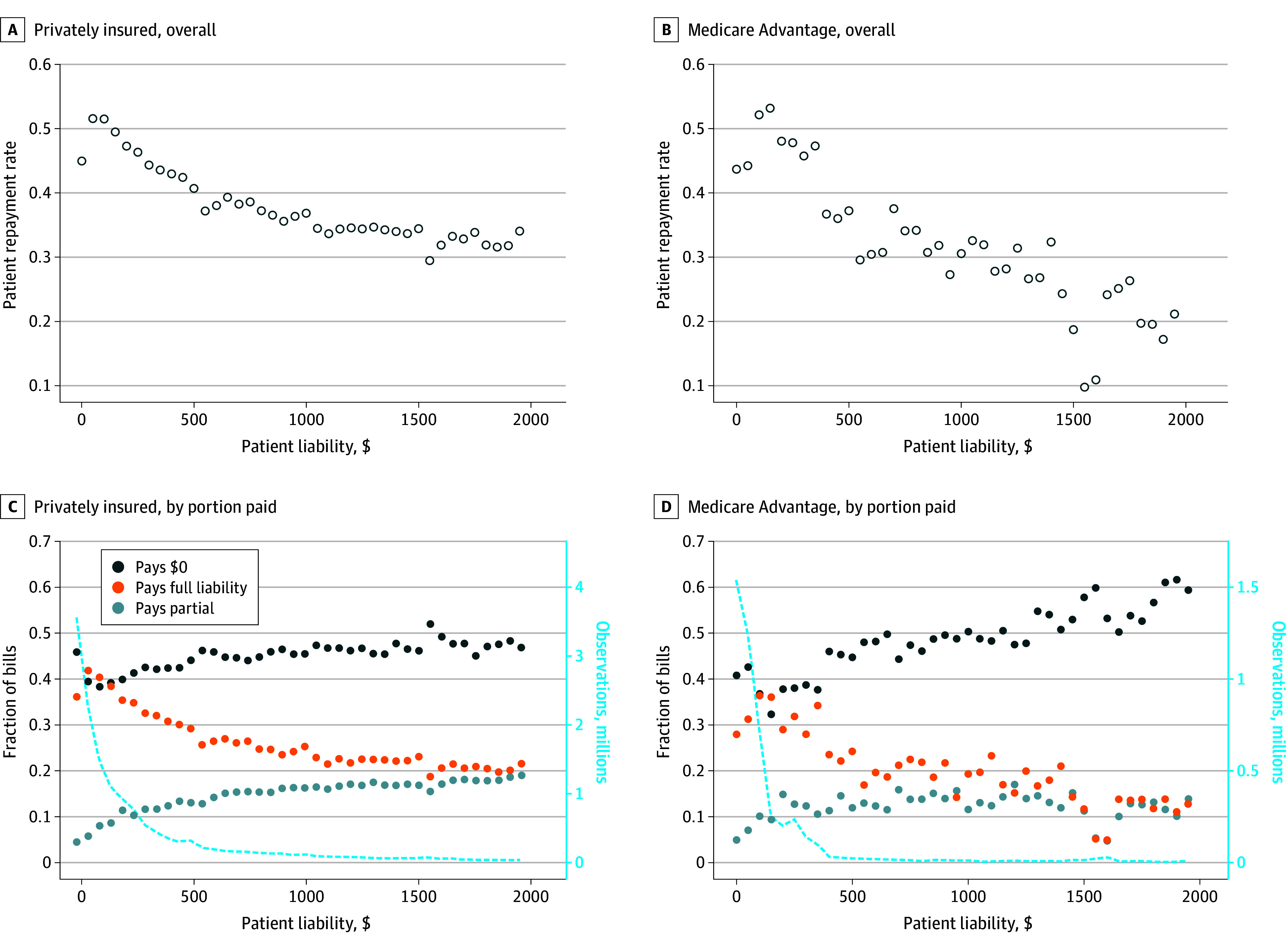
Repayment Rate by Bill Size, 2023 This analysis includes bills under $2000 (sample sizes decrease substantially with higher amounts) from visits or discharges in 2023. Figure illustrates mean repayment rate for bills in $50 buckets. Sample restricted to episodes with positive patient liability. See eAppendix in Supplement 1 for details about FinThrive data source and construction.

Repayment rates also varied by bill size. Among bills with liability under $2000 in 2023, repayment rates were generally lower for larger bills (Figure 4). Among the privately insured, repayment rates for bills over $1000 were typically below 35%, compared with approximately 50% for bills that were approximately $100. This pattern is similar among Medicare Advantage enrollees, though repayments rates were even lower for larger bills.

We observed an exception to this trend among the smallest bills in our sample. Among those with private insurance, repayment rates were lower for bills under $50 (45.0%) than for bills between $50 and $299 (which had mean repayment rates ranging from 51.6% to 46.3%). Similarly, among those on Medicare Advantage plans, repayment rates were lower for bills under $100 (ranging from 43.6% to 44.2%) than for bills between $100 and $399 (ranging from 52.1% to 47.3%). Additionally, we observed higher repayment rates for outpatient episodes than inpatient episodes, which at least partially reflects smaller bill sizes (eAppendix in Supplement 1). This result is similar across years (results not shown).

Finally, we illustrated unpaid liabilities as a fraction of total expected payments from insurers and patients (eAppendix in Supplement 1). Across all years, mean (SD) unpaid patient liability was 4.0% (0.8%) and 2.6% (0.6%) of total expected payment for inpatient episodes (unweighted) for privately insured and MA patients, respectively. Among outpatient episodes, unpaid liability represented a mean (SD) of 7.2% (1.1%) and 4.3% (1.0%) of total expected payments for patients with private insurance and MA, respectively. These rates of unpaid liabilities increased slightly over our sample period.

## Discussion

In this cross-sectional study of 2018 to 2024 hospital revenue cycle management data, patient repayment of cost sharing owed is incomplete and has fallen in recent years. This result remains similar if we measure repayment rate within 1 year of a visit. Our results also show that the mean repayment rate describes almost no patient’s experience. Instead, almost all patients pay their entire liability or none of it.

The recent decline could reflect recent changes to the treatment of medical debt that may increase the rate of unpaid bills. However, our empirical setting does not allow us to formally test that theory. While the number of privately insured enrollees in our sample is relatively constant in recent years, it is possible that changes to the composition of this group may contribute to observed trends.

High prices may also contribute to incomplete repayment rates by increasing some bills directly or triggering greater cost sharing to offset premium increases. That said, we observe incomplete repayment rates among Medicare Advantage enrollees who face lower prices.

In theory, repayment rates associated with bills from 2022 or 2023 could increase if an unusually large share of patient liability is paid multiple years after care; however, that appears highly unlikely given trends we observe from prior years. Even if so, very long delays in repayment would represent a substantial cost to hospitals and doctors.

Perhaps surprisingly, repayment rates for the smallest bills in our sample were lower than modest bills. This may reflect the administrative burden associated with collection activities, which may not be worth it for the smallest bills. It is also possible that consumer attentiveness is higher for more substantial bills.

The sharp rise in patient liability observed in January of each year, particularly among the privately insured, illustrates the effects of deductibles resetting each calendar year. As a result, patients have unusually high liability early in the year compared with a plan that used more consistent copayments or coinsurance for all visits (even holding overall financial liability constant). Notably, however, we do not see any major differences in repayment rates during periods of higher and lower patient liability. This suggests that hospitals and physicians will ultimately recoup lower aggregate payments for otherwise similar visits that occur early in the year compared with those that occur later, when patients owe a smaller portion of the bill.

While unpaid patient liability clearly represents a cost to hospitals and physicians, the implications for patients are less obvious. Particularly if consumers have competing financial obligations, avoiding repayment of cost sharing may free up money for other uses. That may come with costs like the inability to receive future care from the same hospital or physician, legal action, or having bills sent to collections. As noted earlier, though, the direct costs associated with the latter has declined in recent years.

Though unpaid liability currently represents a modest portion of expected payments, these trends may trigger reactions by hospitals and physicians. For example, they may seek more payment ahead of service when feasible. Though, the Emergency Medical Treatment & Labor Act requires hospitals to treat patients in emergencies, regardless of the ability to pay, thereby limiting the use of prepayment in some cases. It is also possible that incomplete repayment rates may affect negotiations between hospitals or physicians and health insurers, particularly if unpaid liabilities grow (eg, seeking greater negotiated rates from insurers that tend to have higher cost sharing).

These trends also highlight the inadequacy of claims data to paint a complete picture of clinician revenue. Analyses of claims data tend to assume that patient cost sharing is paid in full, while our results indicate that is clearly not the case. Thus, claims-based analyses of metrics such as hospital prices and spending are incomplete measures of actual collected and paid amounts.

### Limitations

This study has limitations. First, findings may not be generalizable beyond the sample of hospitals in this study, particularly if hospitals engaged with FinThrive differ from other hospitals. Second, this study does not capture the full scope of bills involved in each episode, as the sample includes hospital facility services and professional services billed through the hospital but excludes bills from other professionals and stand-alone facilities that do not bill through a hospital. Additionally, our analysis does not include bills that are sold to collections agencies; however, these bills are rarely paid. For example, less than 4% of medical collections included on credit reports are ever reported as paid.^11^

## Conclusions

Patient cost sharing repayment rates have fallen in recent years, resulting in both medical debts and collections shortfalls for hospitals and clinicians. Observed declines in patient repayment rates coincide with consumer protections removing medical debt from credit reporting but may reflect multiple factors. If declines in cost sharing repayment continue, hospitals and clinicians may increasingly seek payment ahead of service, when allowable.

## Notes

Supplement 1. **eAppendix.** Online Supplement

Supplement 2.
Data Sharing Statement
