# Supplementary material for: Patient Repayment of US Hospital Bills From 2018 to 2024
Source: JAMA Health Forum. 2025 Aug 8;6(8):e252284. doi: 10.1001/jamahealthforum.2025.2284 (PMC12334956; doi:10.1001/jamahealthforum.2025.2284)
Supplement: Supplement 1. — eAppendix. Online Supplement [file jamahealthforum-e252284-s001.pdf]

Supplemental Online Content

Ippolito B, Trish E, Duffy EL, Vabson B. Patient repayment of US hospital bills from 2018 to 2024. *JAMA Health Forum.* Published online August 8, 2025. doi:10.1001/jamahealthforum.2025.2284

eAppendix. Online Supplement

This supplemental material has been provided by the authors to give readers additonal information about their work.

© 2025 Ippolito B et al. *JAMA Health Forum*.

eAppendix. Online Supplement

Section 1. Data construction
Our data come from FinThrive—a large revenue cycle management company. The data cover inpatient discharges and outpatient visits which occurred between the first quarter of 2018 and the third quarter of 2024. Before additional restrictions, the full sample includes observations with information about patient liability and payments from 494 hospitals across 27 states.

We drop observations with patient liability over $1 million (notably, this excludes a single observation with a data entry error resulting in patient liability of over $700 million). In roughly one percent of cases, we observe payments from a secondary insurer that are equal to the exact amount of patient liability and observe zero payments from patients. In this small number of cases, we assume remaining patient liability was zero. Our analysis implicitly focuses on only those patients who faced non-zero liability.

We drop all observations from hospitals reporting implausible payment data, which we conservatively define as hospitals with average repayment rates below 5 percent. Across our entire sample, this excludes 853,803 observations. We drop any remaining observations where one of our calculated repayment rates is negative or greater than one, since these reflect data errors. Across our entire sample, this excludes just over 1.3 million observations. In total, these data restrictions eliminate 6.6% of our starting sample and leaves us with just over 30.7 million observations.

eFigure 1: Sample Restrictions for Primary Analysis Sample

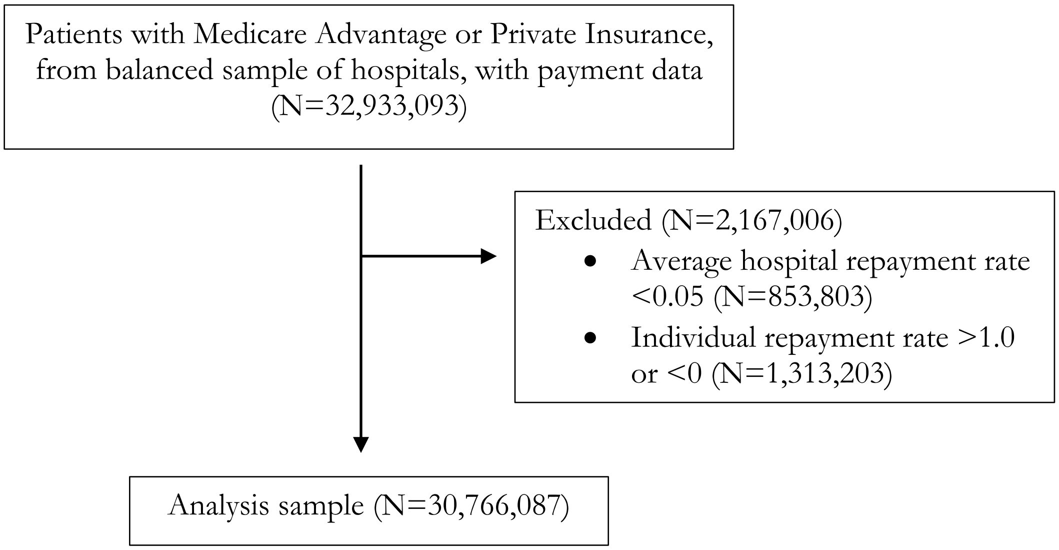

These data do not explicitly categorize those enrolled in Traditional Medicare versus Medicare Advantage. However, they do include detailed descriptions of plan and payer types, which allows us to manually construct these categories. We use these data to identify enrollees in Traditional Medicare and Medicare Advantage. Specifically, those with Medicare as their primary payer are classified into Traditional Medicare or Medicare Advantage if their detailed payer or plan description

© 2025 Ippolito B et al. *JAMA Health Forum.*

include any of the following phrases (ignoring any capitalizations). This process classifies the vast majority of Medicare enrollees (e.g., in 2019, this classifies 89 percent of observations with Medicare as a primary payer). Our analysis excludes any Medicare Advantage enrollees who report being dually eligible for Medicaid since they face trivial cost sharing liability.

eTable 1: Search Words Used to Identify Medicare Plan Type

**Traditional Medicare**	"medicare" "medicare a/b" "medicare ab" "medicare acute" "medicare ip" "medicare medical ins" "medicare part a" "medicare part a b" "medicare part a and b" "medicare part b" "medicare part b only" "medicare pffs" "medicare plan b" "medicare ffs" "medicare-ffs" "medicare a & b" "medicare part a-b" "medicare a" "medicare a and b" "medicare-ffs" "traditional medicare" "medicare part a b" "medicare plan a" "medicare outpatient" "medicare (inpatient only)" "medicare op" "medicare a and b in/out" "medicare part a and b outpatient" "medicare (a)" "medicare op/er" "medicare - ffs" "medicare outpatient without adjustments" "medicare out-patient part b"
**Medicare Advantage**	"advantage" "aetna" "anthem" "bcbs" "blue" "cigna" "humana" "molina" "uhc" "united" "wellcare" "hmo" "ppo" "managed" "ma" "wellmed"

As noted in the main text, our primary sample includes a mean of 355.5 thousand (private insurance) and 90 thousand (Medicare Advantage) observations per month after all data restrictions. If we included patient who did not face liability, monthly sample sizes averaged 503 thousand and 146.1 thousand for private and Medicare Advantage, respectively. Given that we use a balanced sample of hospitals that appear in all quarters of data, it is unsurprising that we observe a relatively similar number of observations in each quarter. As expected, however, we observe a sharp decline in discharges in early 2020 due to the COVID-19 pandemic. That decline was relatively brief; episodes of healthcare utilization in our sample reached close to pre-pandemic levels by the summer of 2020.

© 2025 Ippolito B et al. *JAMA Health Forum*.

eFigure 2: Number of Observations with Positive Patient Liability by Month And Type of Insurance, 2018-2023

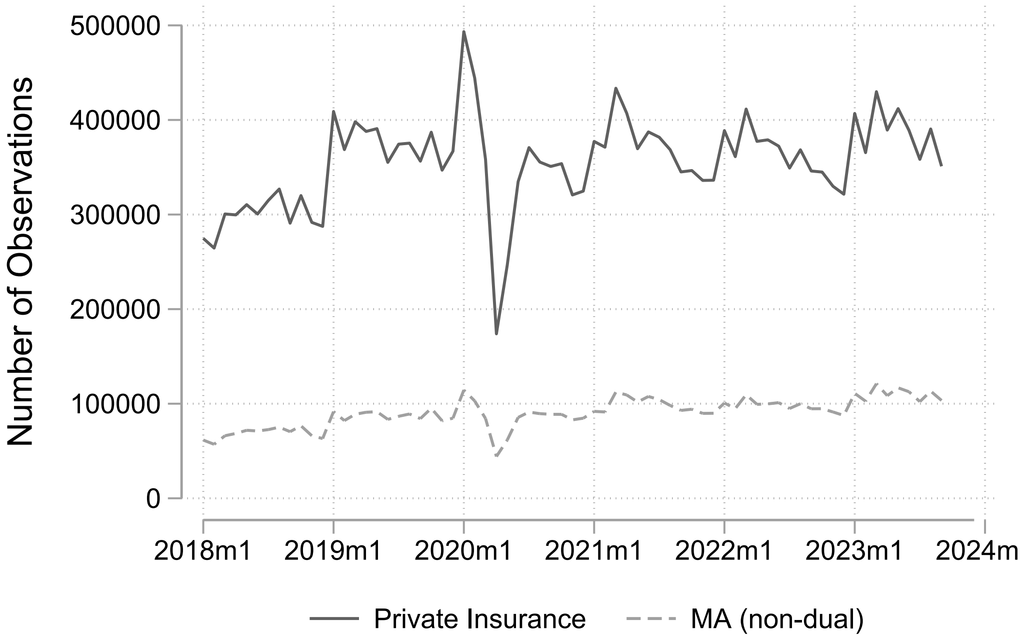

[a] Analysis of FinThrive data. This illustrates the number of observations by month. Medicare Advantage category excludes those with dual eligibility for Medicaid.

© 2025 Ippolito B et al. *JAMA Health Forum*.

Section 2. Sample characteristics

In the main text, we compare characteristics of hospitals in our sample to the universe of hospitals in the country. That comparison weights each hospital equally. In eTable 2 we weight each hospital in our sample by the number of episodes observed in our data. When calculating the percentages in each category for the overall U.S. we weighted by the number of discharges.

Under episode level weighting, our sample of hospitals has a very similar share of urban hospitals to the U.S. average. We observe a smaller share of visits at teaching hospitals than the U.S. average. Finally, while we observe more observations from large hospitals than the U.S. average, this difference is smaller than with equal weighting.

eTable 2. Sample Characteristics, Billing Episode Level

Characteristic	Balanced Sample[a] N=217	Full Sample[a] N=399	U.S. Sample (discharge weighted percentages)[b] N=6,152
Urban	28,384,630 (92.26%)	38,631,145 (91.77%)	4,154 (92.85%)
Teaching[c]	12,244,128 (39.80%)	17,194,816 (40.85%)	1,567 (68.65%)
Bed Size			
Under 200	3,171,777 (10.31%)	4,566,943 (10.85%)	4,742 (29.43%)
200-299	7,870,726 (25.58%)	10,123,346 (24.05%)	505 (16.25%)
300-499	5,096,918 (16.57%)	7,183,661 (17.06%)	478 (24.21%)
500+	14,543,484 (47.28%)	20,123,192 (47.80%)	292 (30.11%)
Missing	83,182 (0.27%)	100,067 (0.24%)	104 (0.00%)

[a] Analysis of hospitals from FinThrive data.

[b] Data from RAND Hospital Files for calendar year 2023 (data vintage February 2025), which are based on the Healthcare Cost Report Information System from the Centers for Medicare and Medicaid Services. Urban status for this sample is defined as a Rural-Urban Continuum Code greater than three. Counts are unweighted hospital counts, and percentages are weighted by the number of discharges reported for each hospital.

[c] Teaching hospitals defined as those who are either members of the Council of Teaching Hospitals or have residency programs.

Section 3. Average patient liability, comparing inpatient and outpatient

In the main text, we illustrate mean patient liability among those with private and Medicare advantage insurance over time. In this section, we separately graph these trends for inpatient visits and outpatient visits. This allows us to confirm that higher patient liability among the privately insured is not due to a larger share of inpatient bills. Instead, privately insured individuals face meaningfully higher cost sharing in both settings.

© 2025 Ippolito B et al. *JAMA Health Forum*.

eFigure 3: Patient Liability per Capita, by Type of Insurance and Visit Type[a]

Panel A: Inpatient

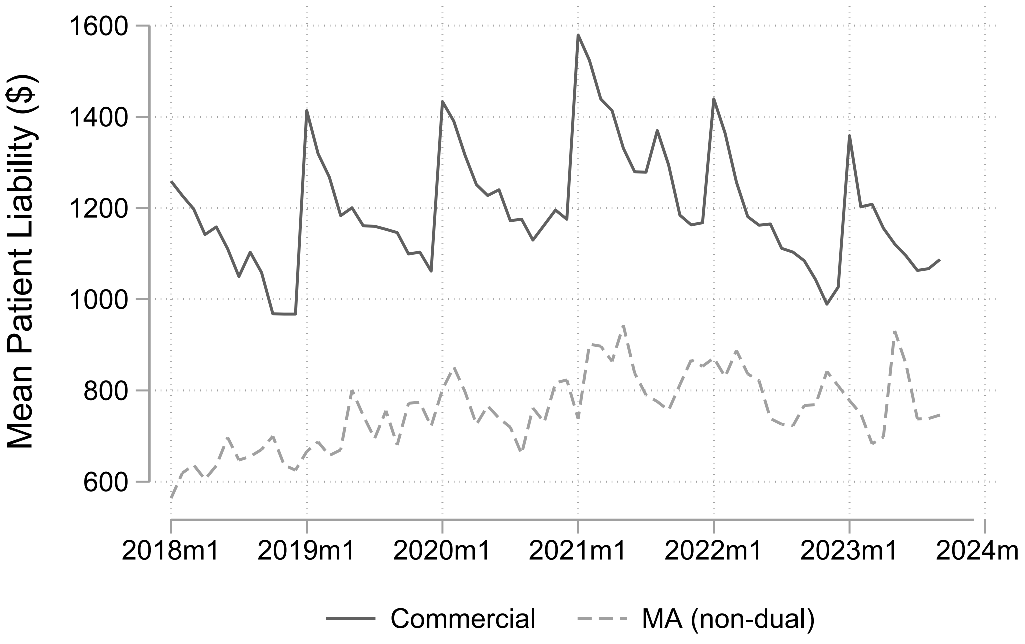

Panel B: Outpatient

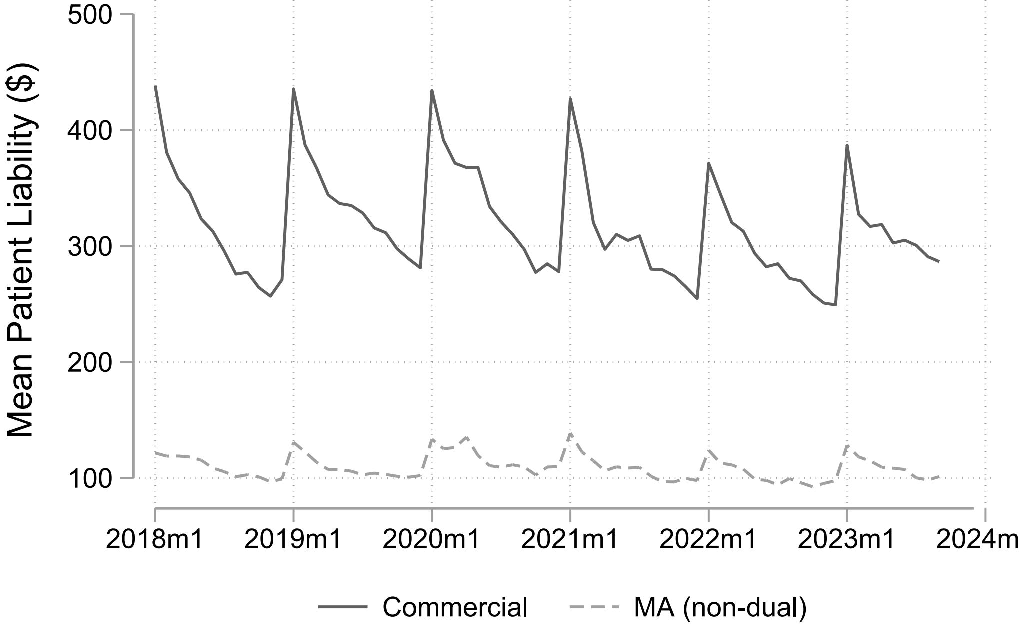

[a] Analysis of FinThrive data. This illustrates the mean liability among individuals, including those with no liability. Medicare Advantage (MA) category excludes those with dual eligibility for Medicaid.

© 2025 Ippolito B et al. *JAMA Health Forum*.

Section 4: Results when using unbalanced sample of hospitals

Our primary analysis uses observations from hospitals that appear in every quarter of our sample. This alleviates the concern that changes in repayment rates over time could be driven by hospitals moving in and out of our sample. A changing sample of hospitals could otherwise be consequential because repayment rates appear to vary substantially across hospitals. This reflects differences in patient and service mix, as well hospitals' own effectiveness at collecting patient liability. Holding the set of hospitals in our sample fixed enables us to abstract from these concerns.

For completeness, we illustrate how our primary result varies based on this decision. Panel A of the figure below replicates our primary result from the main text using our balanced panel of 216 hospitals. Panel B includes observations from the 419 hospitals which remain after initial data restrictions. The results are very similar in both samples.

© 2025 Ippolito B et al. *JAMA Health Forum*.

eFigure 4: Repayment Rates Using Balanced and Unbalanced Sample of Hospitals, 2018-2023[a]

Panel A: Balanced Sample

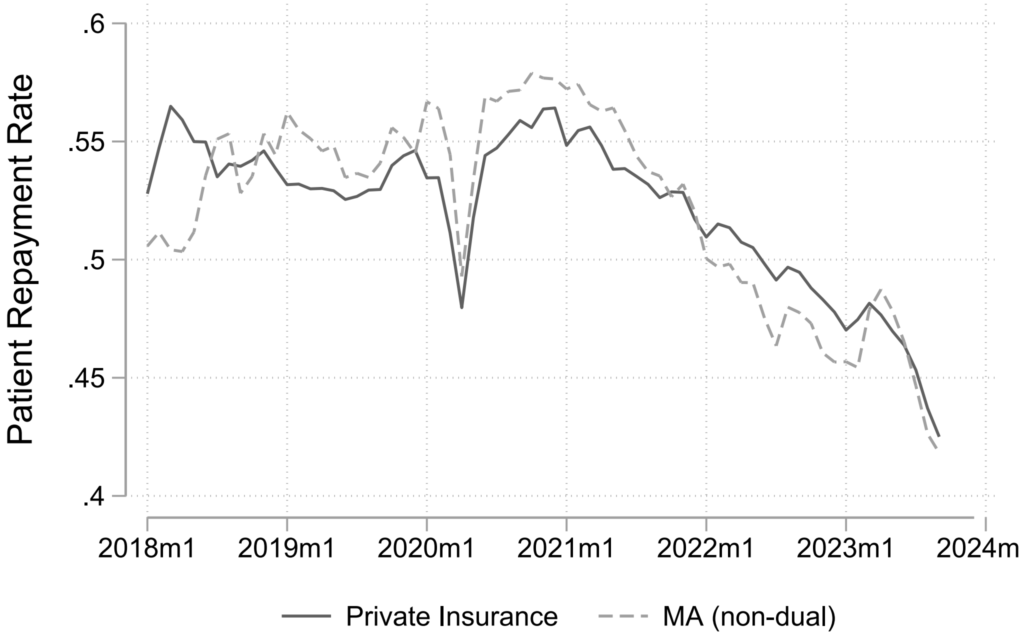

Panel B: Unbalanced Sample (all hospitals)

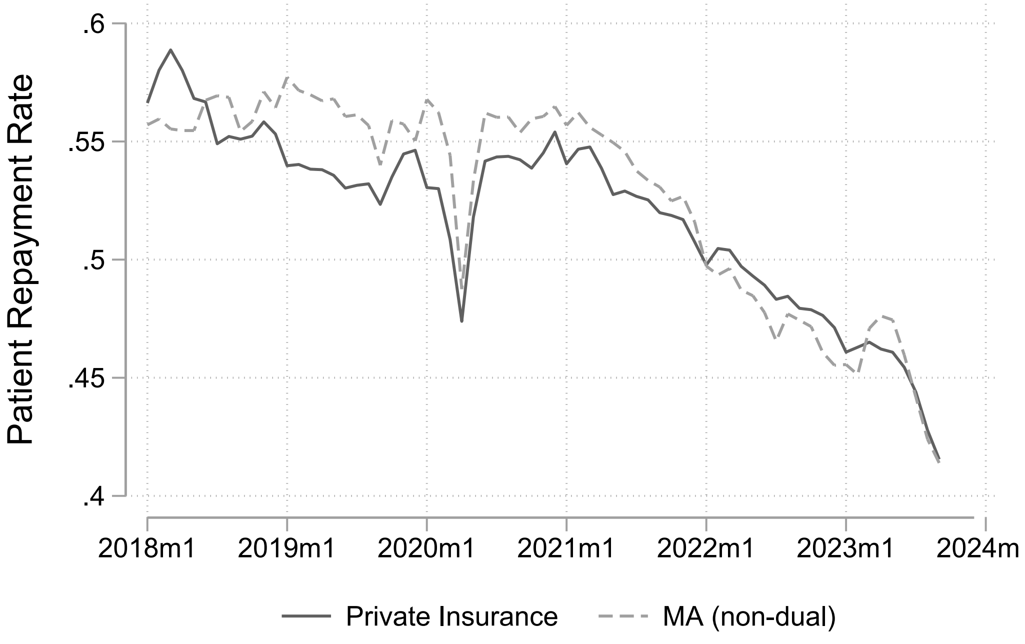

[a] Analysis of FinThrive data. Medicare Advantage (MA) category excludes those with dual eligibility for Medicaid. Balanced panel includes only observations from hospitals observed in every quarter of our data. Unbalanced panel includes observations from hospitals regardless of how often they appear in our data.

© 2025 Ippolito B et al. *JAMA Health Forum*.

Section 5. Exploring alternative repayment rate measures

Our main analysis defines the repayment rate as the fraction of patient liability paid as of September 2024 for all bills regardless of discharge date. We restrict our primary sample to discharges that occurred no later than the third quarter of 2023 which allows at least one year for patients to pay any outstanding liabilities. However, discharges from earlier years still have had a longer time to be paid than discharges in more recent years. This raises the possibility that declining repayment rates observed in the later part of our sample may be due to this censoring of the data. In other words, repayment rates in 2023 may be lower than in earlier years only because patients have had less time to pay those bills. In this section, we use a secondary data file to explore this issue and find that our core result is similar if we use an alternative definition of repayment rates that alleviates this concern.

Our primary analysis of patient liability and payments uses episode-level data. These data are provided by hospitals and include all bills and payments associated with a visit, including those on the insurer side as well as (separately) on the patient side. FinThrive accesses these data from hospitals' accounting and revenue cycle management systems. Given that the format of this data may vary somewhat across hospitals, FinThrive must harmonize and standardize the data when producing a consolidated file.

In addition to these data, FinThrive also produces more detailed transaction-level data for a subset of observations. These data include information on the timing and amount of every individual transaction associated with an episode, including bills and payments on the insurer and patient sides. A single episode can be associated with multiple insurer bills (since a single hospital visit can lead to multiple separate claims filed) and multiple patient bills. This data also tracks the full insurer adjudication cycle between the hospital and the insurer, including the initial bill amount from the hospital, any contractual adjustments by the insurer, as well as any other back and forth due to denials or adjustments. The timing of each transaction is recorded in days relative to the discharge or visit date. These data allow us to measure what fraction of patient liability is paid within one year for each visit. While this omits some payments that occur later, it provides us a measure that is consistent across all discharge dates.

Each transaction is assigned to a specific category (i.e., insurer payment, patient payment) by FinThrive. FinThrive manually derived this field from text-based description fields from the original hospital data, which varied substantially from hospital to hospital. Consequently, the classification process is imperfect. FinThrive was unable to classify the transaction in about 20 percent of cases. These unclassified observations are heavily concentrated at a subset of hospitals. For this analysis, we drop all hospitals where most transactions are unclassified.

To do so, we calculate the fraction of transactions recorded as "unknown" for each hospital. We also calculate how often the sum of patient payments in these transaction files equal the aggregate patient payments recorded in our primary dataset. High rates of "unknown" transactions or low match rates between datasets are both indications that a hospital has recorded data in a way that prevents successfully classifying transactions.

Transaction-level data are available for 209 of the hospitals which are included in our primary analysis sample, for which we observe 4,761 hospital-quarter observations. Below, we illustrate the distribution of each metric across hospital-quarter observations. In both cases we see that a small number of hospital-quarter observations have very high rates of data errors—cases where either

patient payments never match between the two files (panel A) or where all observations are recorded as “unknown” (panel B).

© 2025 Ippolito B et al. *JAMA Health Forum*.

eFigure 5: Investigating Data Errors in Transaction Files[a]

Panel A: Match Rate for Patient Payments Between Data Files

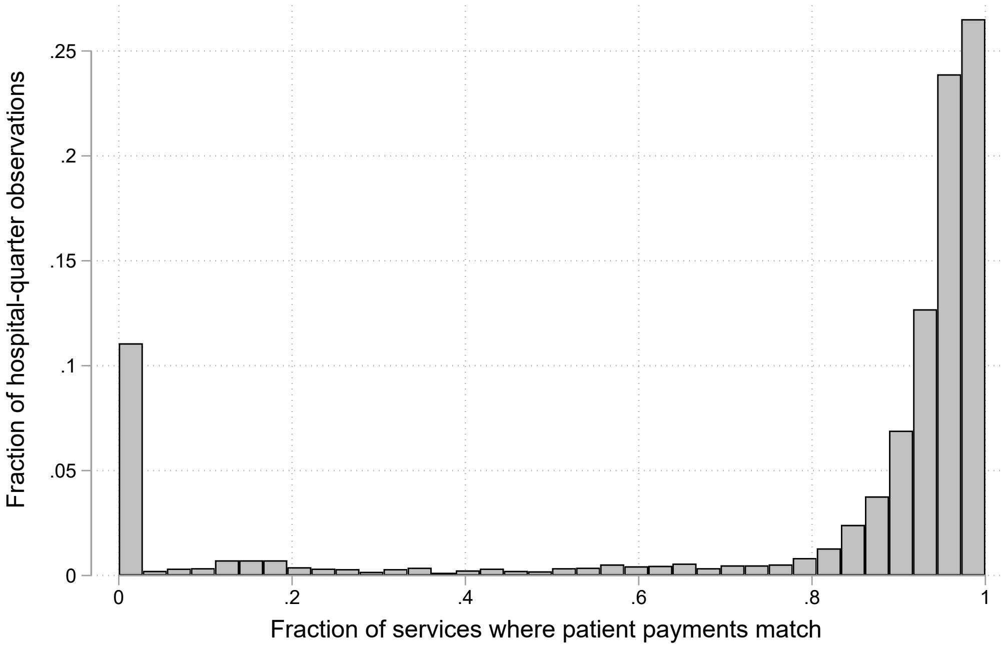

Panel B: Rate of "Unknown" Transactions

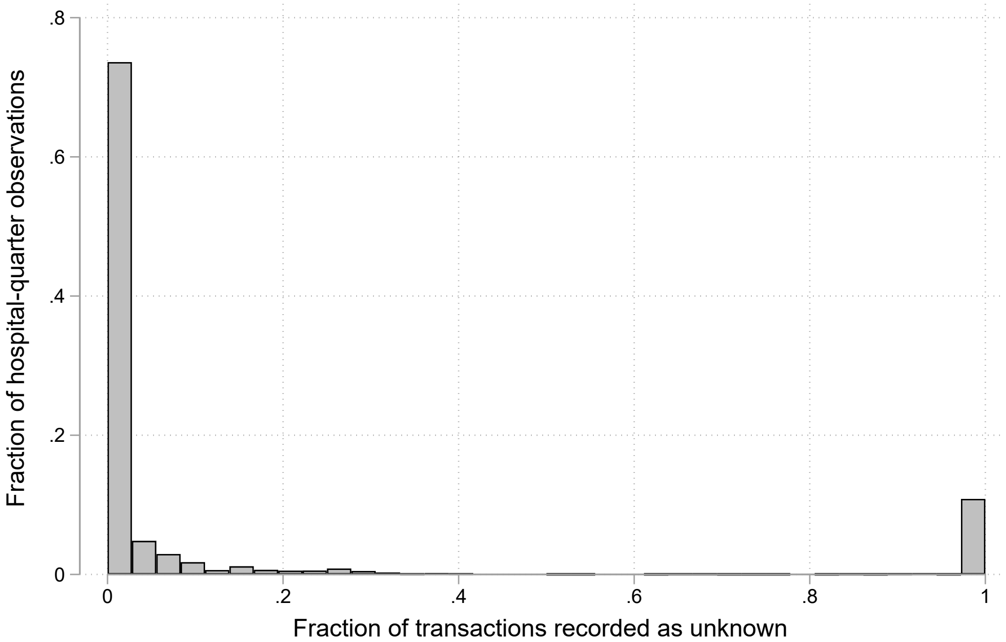

[a] Analysis of FinThrive data. Panel A plots the fraction of hospital-quarter observations where patient payments in transaction data match patient payments in our primary data. Panel B plots the fraction of transactions recorded as "unknown" for each hospital-quarter.

© 2025 Ippolito B et al. *JAMA Health Forum*.

To be conservative, we exclude hospitals from this analysis if they ever have a quarter where more than 10 percent of observations are recorded as "unknown" or where patient payments do not match across files at least 10 percent of the time. As the figure above illustrates, this predominantly excludes hospitals which have quarters where the majority of transactions cannot be effectively categorized by FinThrive. This is consistent with some institutions recording data in a way that systematically undermines attempts to classify this more granular data. After this conservative restriction, we are left with 116 hospitals which have reliable transaction-level data in all quarters of our data.

Below, we illustrate repayment rates among this sample using alternative definitions. Our goal is to compare repayment rates under two different definitions—one that includes patient payments made at any time though September 2024 and one that includes only payments made within one year of discharge. The difference between these lines will illustrate how important this censoring is to the trend we observe. We expect the difference to be larger in the early part of our sample because those bills have had the longest to be paid and vice versa for more recent bills.

Below, we illustrate our overall result from the main text using the full sample of privately insured individuals. We then calculate repayment rates for our matched sample where we observe more granular transaction-level data. First, we replicate the primary result using episode-level data for this subset of our population. Repayment rates for this subset are a couple percentage points higher than the full sample but follow a very similar trend over time. Second, we calculate repayment rates using transaction-level data, including payments at any time through September 2024. Repayment rates are slightly lower across all years, primarily reflecting the fact that a small number of transactions are misassigned or unclassified. Reassuringly, the trend observed using these data is nearly identical to what we observe using episode-level data. Finally, we replicate this timeseries but only include patient payments made within one year of discharge.

As expected, excluding payments made after one year slightly lowers the observed repayment rate. This difference is larger in earlier years of the sample and is effectively zero by the end of our sample. This reflects the fact that the most recent bills included in our analysis have only had roughly one year to be paid absent any data restrictions. We see broadly similar patterns among those included in Medicare Advantage.

Across our entire sample, restricting our analysis to only payments made within one year of discharge lowers repayment rates by 2.4 and 1.4 percentage points for those on private insurance and MA, respectively. These differences rise to 2.8 and 1.7 if we exclude 2022 and 2023, where this restriction is mechanically less important.

Taken together, these data suggest that this data censoring concern only slightly affects our primary results and that effect is most pronounced in the early years of our sample. The reduction in payment rates in more recent years remains similar even after directly accounting for this potential concern.

© 2025 Ippolito B et al. *JAMA Health Forum*.

eFigure 6: Patient Repayment Rates Within One Year of Discharge/Date of Service[a]

Panel A: Privately Insured

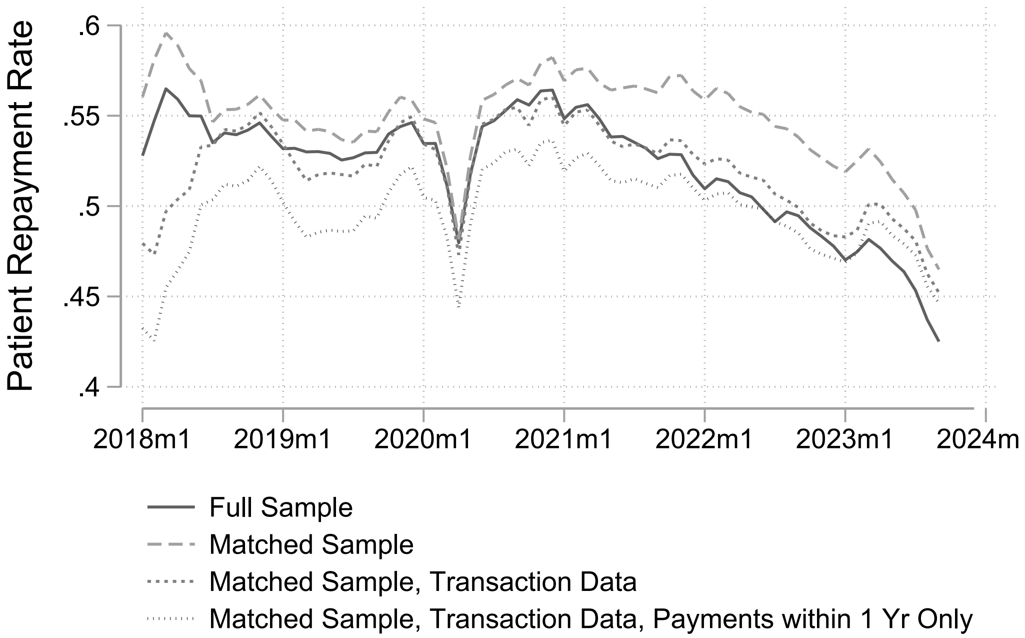

Panel B: Medicare Advantage

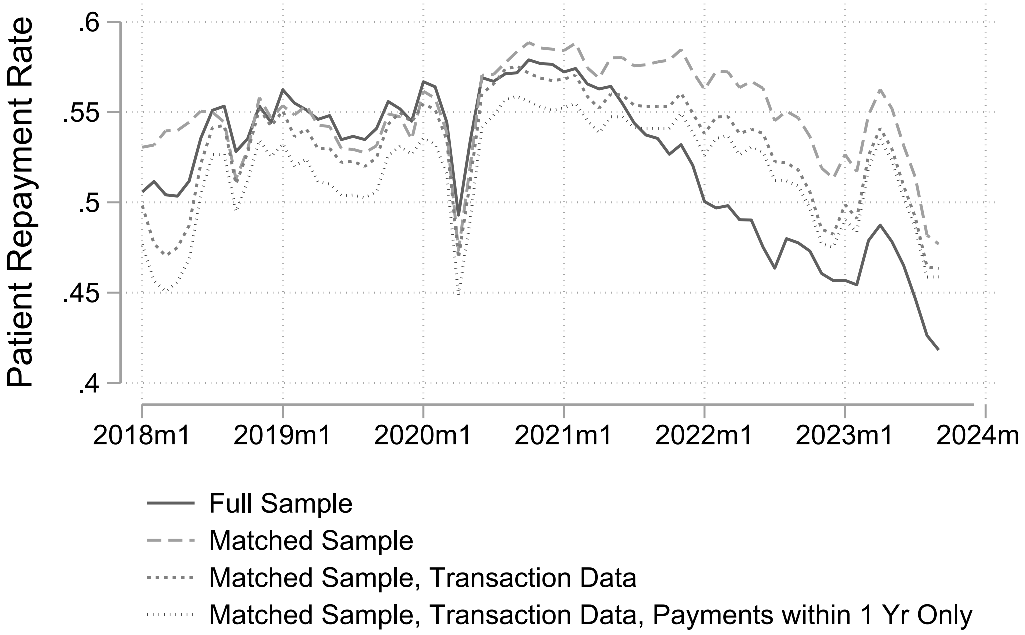

[a]Analysis of FinThrive data.

Section 6: Repayment rates by age

© 2025 Ippolito B et al. *JAMA Health Forum*.

The overall repayment rates illustrated in the main text include all patients within either private insurance of Medicare Advantage within a given month. However, repayment rates vary considerably across ages within these groups—particularly for privately insured patients.

In the figure below we illustrate repayment rates by age for each payer type. For simplicity, we include data from the first quarter of each year from 2018 through 2023. When graphing results for the privately insured population we include only those under age 65 to abstract from complications associated with multiple insurance types after Medicare eligibility. Similarly, we only graph those on Medicare Advantage who are over 65 because the population of enrollees under this age is small and meaningfully different.

These data show that repayment rates vary significantly by age, particularly among the privately insured. Repayment rates are lowest for those in their 20s and increase with age among adults. Repayment rates for individuals under 18 are similar to those of middle-aged adults—a result that is unsurprising given that parents responsible for payment likely come from that population. This overall pattern is consistent with prior research which showed medical collections were most common among young adults and decreased with subsequent ages.[1] Payment rates are less variable by age among those with Medicare Advantage. These data also show that the reduction in repayment rates over time is relatively consistent across ages, particularly for the privately insured population.

[1] Michael Batty, Christa Gibbs, and Benedic Ippolito. Unlike Medical Spending, Medical Bills In Collections Decrease With Patients' Age. Health Affairs 2018 37:8, 1257-1264URL: https://www.healthaffairs.org/doi/full/10.1377/hlthaff.2018.0349

© 2025 Ippolito B et al. *JAMA Health Forum*.

eFigure 7: Repayment Rates by Age

Panel A: Private insurance

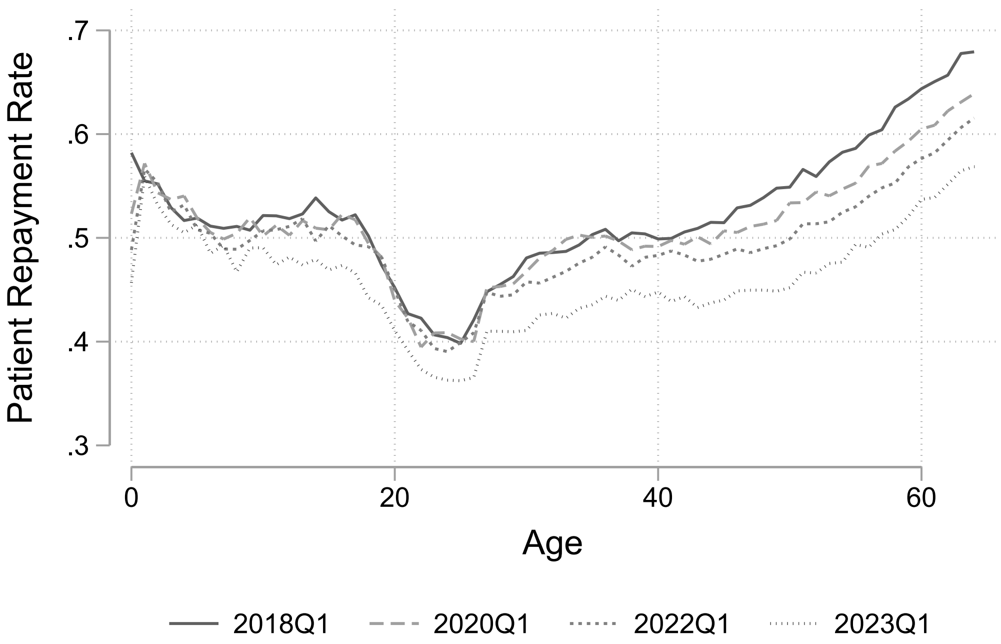

Panel B: Medicare Advantage

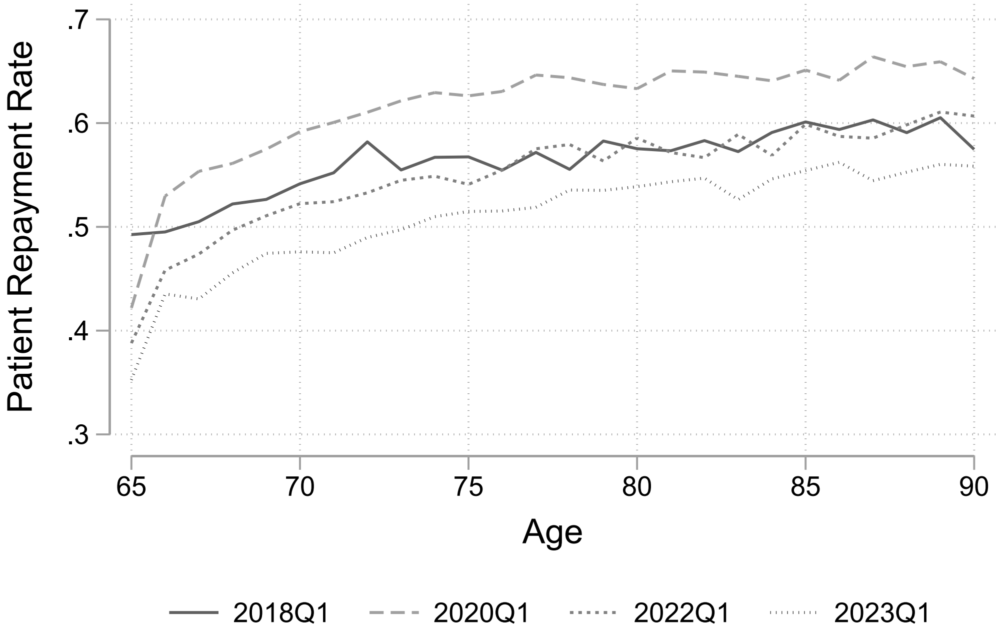

[a] Analysis of FinThrive data. Panel A excludes those over 65 with private insurance as their primary insurance. Similarly, Panel B excludes those under 65 with Medicare as their primary insurance.

© 2025 Ippolito B et al. *JAMA Health Forum*.

Section 7:
Our primary analysis of repayment rates includes bills associated with inpatient (IP) and outpatient (OP) visits. In this section, we illustrate repayment rates for each visit type separately. Panel A of eFigure 8 shows the overall repayment rate for each bill type for those with private insurance and Medicare Advantage. Across our entire sample, we observe higher repayment rates for bills associated with outpatient visits than those associated with inpatient visits. This is true for those with private insurance or Medicare Advantage. This remains true if we focus instead on the one-year repayment rate among those with relevant data (Panel B).

Higher repayment rates among outpatient bills at least partly reflects lower average bill size. However, among those with private insurance, we still observe slightly higher repayment rates for outpatient bills of most sizes, but particularly the smallest ones (eFigure 9, panel A). Among those with Medicare Advantage, we also see higher repayment rates for outpatient bills in the smallest category, but we observe the opposite for larger bills. These trends may partly reflect differences in insurance plan design that we cannot observe.

© 2025 Ippolito B et al. *JAMA Health Forum*.

eFigure8: Patient Repayment Rates, Inpatient versus Outpatient, 2018-2023[a]

Panel A: Repayment rate

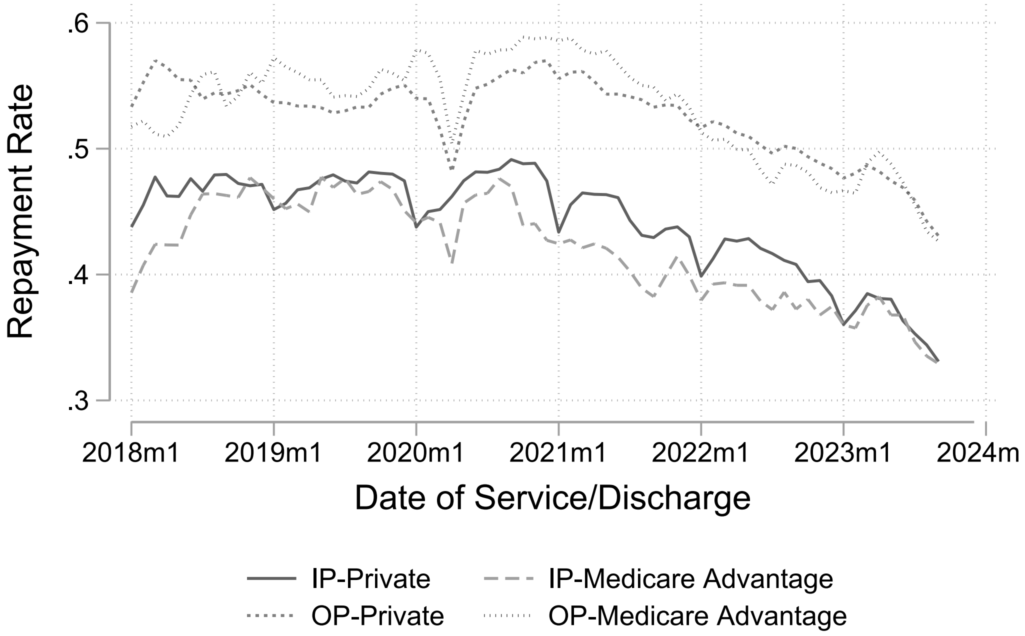

Panel B: One year repayment rate[b]

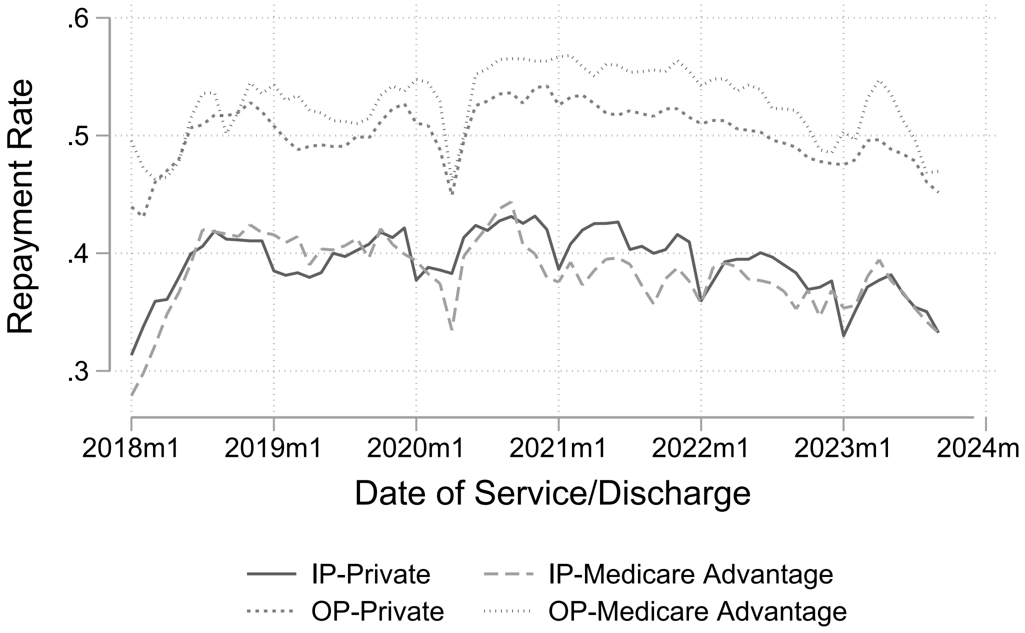

[a] Analysis of FinThrive data. Repayment rate is defined as the fraction of patient liability recorded as paid by September 2024. Medicare Advantage (MA) category excludes those with dual eligibility for Medicaid. Sample restricted to episodes with positive patient liability.

© 2025 Ippolito B et al. *JAMA Health Forum*.

[b] Includes subset of observations with reliable transaction-level information.

© 2025 Ippolito B et al. *JAMA Health Forum*.

eFigure9: Patient Repayment Rate by Bill Size, Inpatient versus Outpatient, 2023[a]

Panel A: Privately Insured

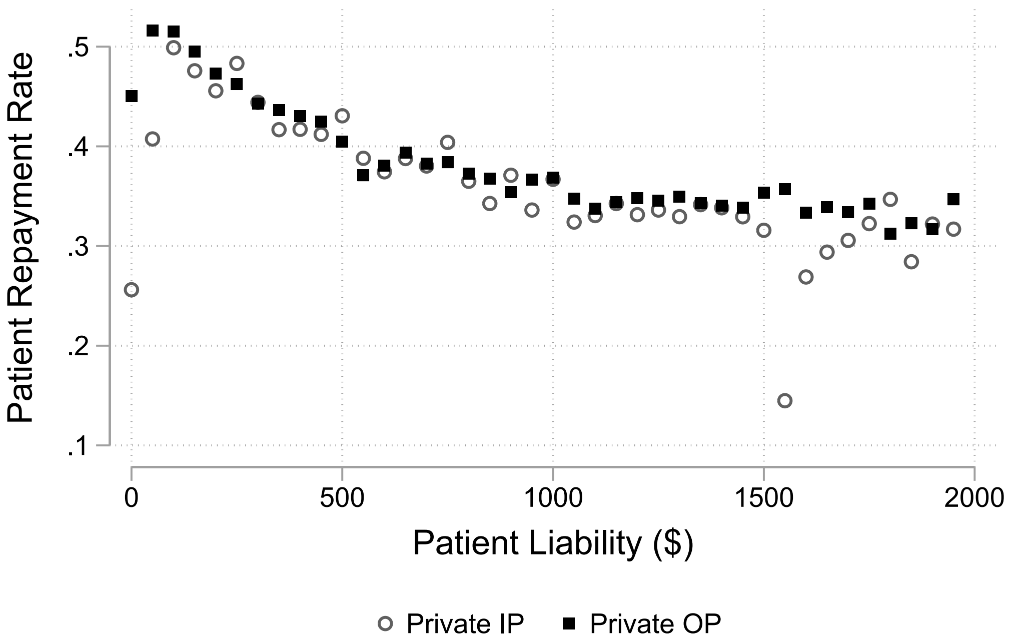

Panel B: Medicare Advantage

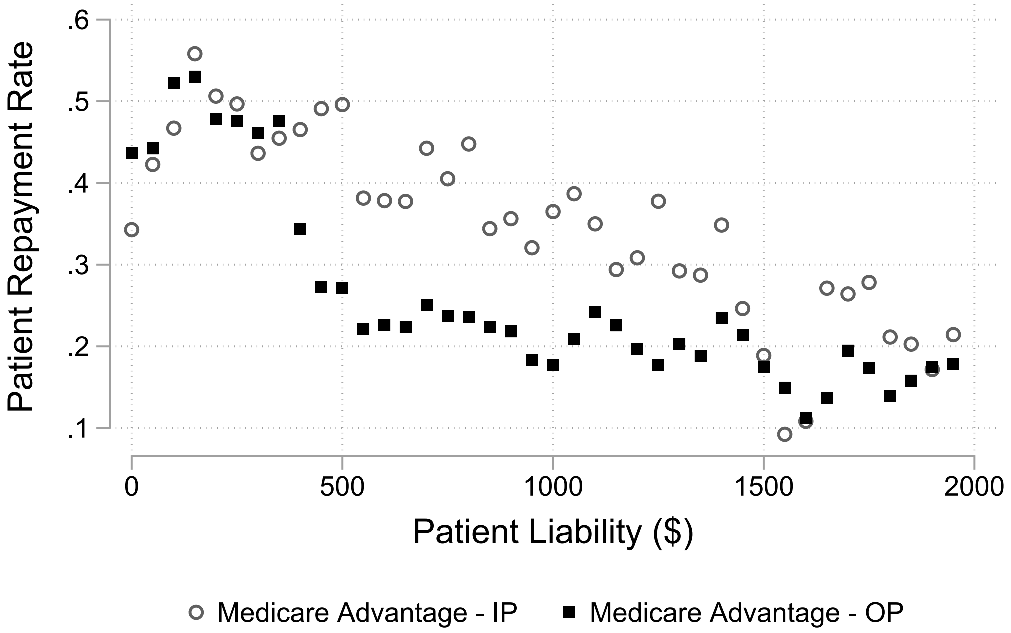

[a] Analysis of FinThrive data. Repayment rate is defined as the fraction of patient liability recorded as paid by September 2024. Medicare Advantage category excludes those with dual eligibility for Medicaid. Sample restricted to episodes with positive patient liability.

© 2025 Ippolito B et al. *JAMA Health Forum*.

Section 8: Unpaid Patient Liability Relative to Total Liability Owed
In the main text, we document the fraction of owed patient liability that is paid. In this section, we put unpaid liability in the context of total expected payments for hospitals or doctors.

First, we calculate patient liability as a fraction of total expected payments, which is equal to the liability owed by patients plus amount owed by insurers. Below, we graph the unweighted mean value for each payer type across our sample (eFigure 8). We calculate this separately for outpatient and inpatient services and for each insurance type. Across our sample, patient liability represents 8.4% and 4.6% of total expected payments for a typical inpatient visit for privately insured and MA patients, respectively. Unpaid liabilities represent 19.0% and 13.3% of total expected payments for outpatient visits, respectively.

Next, we calculate unpaid cost sharing as a fraction of total expected payments from insurers and patients. This measure helps capture how unpaid patient cost sharing affects total expected payment for a typical bill in our sample. Again, we calculate this separately for inpatient and outpatient bills (eFigure 9). Across the full sample, unpaid liabilities account for 4.0% and 2.6% of total expected payment for inpatient bills for privately insured and MA patients, respectively. Among outpatient bills, unpaid liability represented an average of 7.2% and 4.3% of total expected payments for patients with private insurance and MA, respectively. In both cases, unpaid liabilities represent a slightly increasing share of expected payments. This is consistent with recent declines in repayment rates presented in the main text.

© 2025 Ippolito B et al. *JAMA Health Forum.*

eFigure 10: Patient liability as a fraction of total expected payment, 2018-2023[a]

Panel A: Inpatient

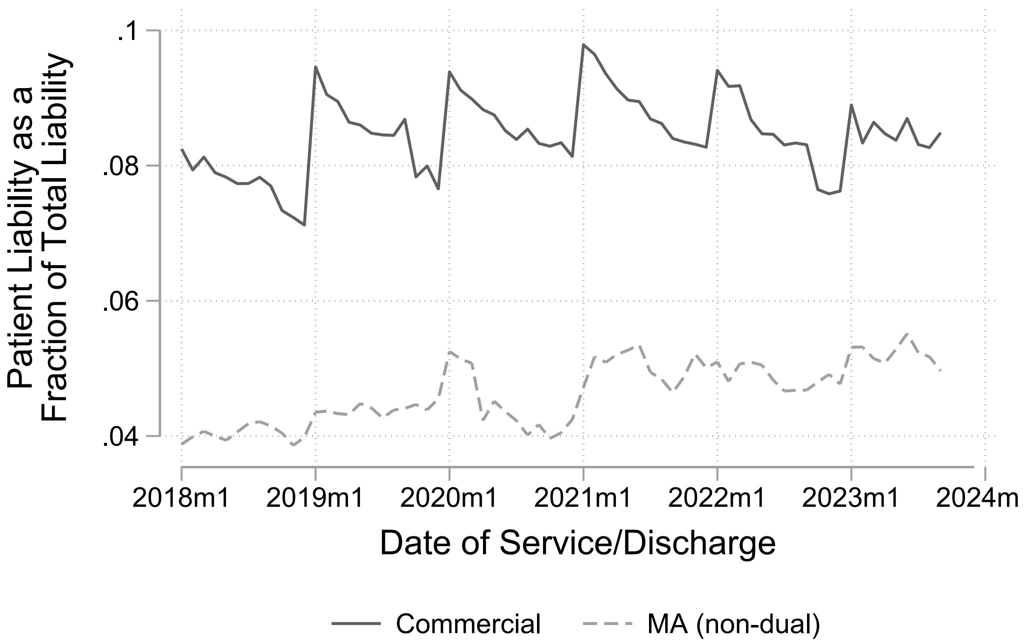

Panel B: Outpatient

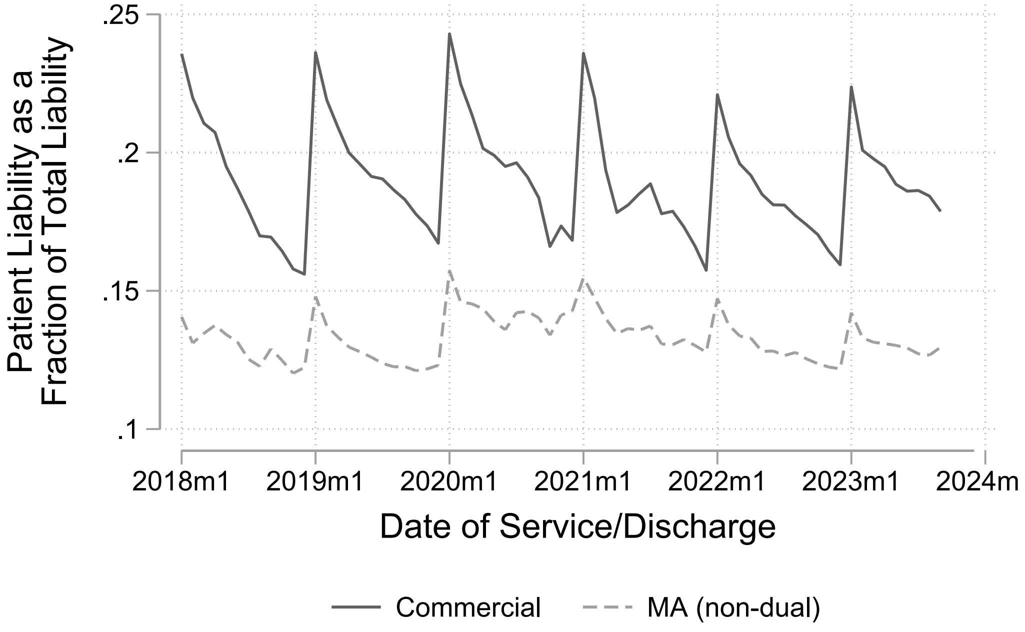

[a] Analysis of FinThrive data. Total expected payments include expected payments (i.e., allowed amounts) by insurers and patients.

© 2025 Ippolito B et al. *JAMA Health Forum*.

eFigure 11: Unpaid patient liability as a fraction of total expected payment, 2018-2023[a]

Panel A: Inpatient

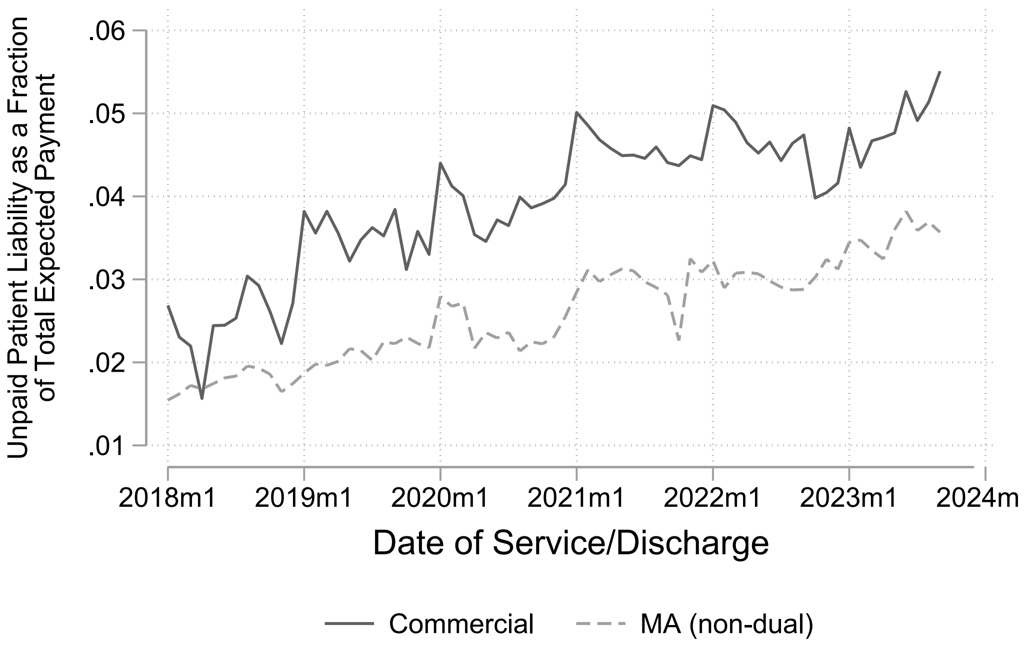

Panel B: Outpatient

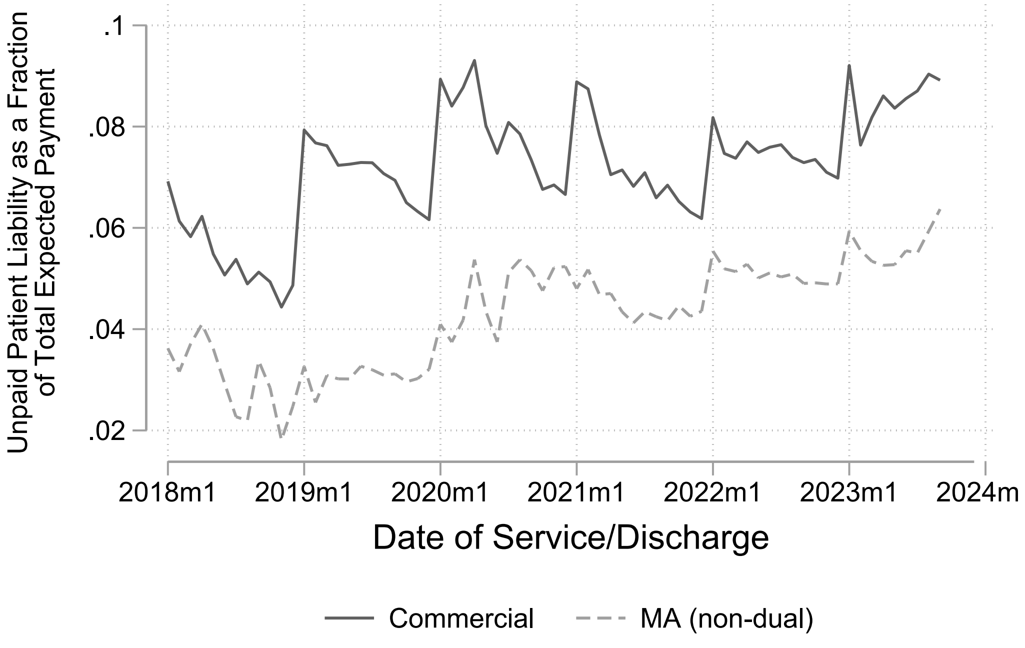

[a] Analysis of FinThrive data. Total expected payments include expected payments (i.e., allowed amounts) by insurers and patients.

© 2025 Ippolito B et al. *JAMA Health Forum*.
