# Supplementary material for: Patient Repayment of US Hospital Bills From 2018 to 2024
Source: JAMA Health Forum. 2025 Aug 8;6(8):e252284. doi: 10.1001/jamahealthforum.2025.2284 (PMC12334956; doi:10.1001/jamahealthforum.2025.2284)
Supplement: Supplement 2. — Data Sharing Statement [file jamahealthforum-e252284-s002.pdf]

Data Sharing Statement

Ippolito. Patient Repayment of US Hospital Bills From 2018 to 2024. *JAMA Health Forum*. Published August 08, 2025. doi:10.1001/jamahealthforum.2025.2284

Data
Data available: No

Additional Information
Explanation for why data not available: The data used in this study is subject to a data use agreement that prohibits us from disclosing the underlying data publicly. We are happy to discuss further if it would be helpful.
